# Single Synonymous Mutations in *KRAS* Cause Transformed Phenotypes in NIH_3_T_3_ Cells

**DOI:** 10.1371/journal.pone.0163272

**Published:** 2016-09-29

**Authors:** Andrew M. Waters, Rachel Bagni, Franklin Portugal, James L. Hartley

**Affiliations:** 1 Cancer Research Technology Program, Frederick National Laboratory for Cancer Research, Leidos Biomedical Research, Inc., Frederick, Maryland, United States of America; 2 Biology Department, Catholic University of America, Washington, District of Columbia, United States of America; Universitat Witten/Herdecke, GERMANY

## Abstract

Synonymous mutations in the *KRAS* gene are clustered at G12, G13, and G60 in human cancers. We constructed 9 stable NIH_3_T_3_ cell lines expressing *KRAS*, each with one of these synonymous mutations. Compared to the negative control cell line expressing the wild type human *KRAS* gene, all the synonymous mutant lines expressed more KRAS protein, grew more rapidly and to higher densities, and were more invasive in multiple assays. Three of the cell lines showed dramatic loss of contact inhibition, were more refractile under phase contrast, and their refractility was greatly reduced by treatment with trametinib. Codon usage at these glycines is highly conserved in *KRAS* compared to *HRAS*, indicating selective pressure. These transformed phenotypes suggest that synonymous mutations found in driver genes such as *KRAS* may play a role in human cancers.

## Introduction

Synonymous mutations are often disregarded because they do not affect the final amino acid sequences of proteins. However, we are learning that codon biases and resulting changes to mRNA secondary structure can alter mRNA stability and ribosomal translation rates [1–5]. Additionally, ribosomal pauses in co-translationally folded proteins can lead to alternative final conformations of a protein with distinct biological outcomes. For example, the *FRQ* gene in the bread mold *Neurospora crassa* contains non-optimal codons that are critical for proper circadian rhythm. Codon optimization of *FRQ* leads to increased FRQ protein levels, altered conformation and phosphorylation changes, and impaired circadian clock function [6,7]. An example in humans is the three-base deletion in the *CFTR* gene that is the most common cause of cystic fibrosis [3]. While historically the loss of the F508 has been the focus of research, recent findings suggest that the synonymous codon change at the adjacent isoleucine 507, and not the deletion of F508, plays the larger role in decreased translation and consequent lack of functional CFTR protein [8]. Additionally, recognition that pairs of synonymous codons are not uniformly distributed in genes has facilitated major advances in vaccine research. Coleman et al. [9] used rare codon pairs to generate engineered poliovirus particles with a modified capsid protein that maintained the wild-type amino acid sequence, and thus immunogenicity, while the infectivity of the particles was decreased by several orders of magnitude. The infectivity of several other viruses has been decreased by similar methods [10, 11]. Moreover, some individuals have a synonymous SNP in their P-glycoprotein (*MDR1*) gene. P-glycoprotein is a broad specificity transmembrane efflux pump responsible for removing many foreign compounds from cells, including chemotherapy drugs. One of these mutations, C3435T, results in a rare isoleucine codon that leads to aberrant folding of the final protein and changes the substrate specificity of the transporter [12].

Synonymous mutations have also been associated with multiple types of cancer. For example, an F17F synonymous mutation is enriched in the pro-survival *BCL2L12* gene in human melanoma samples. This mutation leads to loss of an miRNA binding site and increased mRNA stability, resulting in overexpression of the encoded protein and hyperactivity of anti-apoptotic signaling [13]. p16 is also significantly enriched in synonymous codon changes in melanoma patients compared to the healthy population [14]. Additionally, some genomes contain synonymous mutations at nucleotides adjacent to splice junctions in *TP53*, resulting in aberrantly spliced, inactive p53 transcripts [4]. Synonymous mutations have been reported to act as drivers of human cancers [4] and recently, thousands of “silent drivers” of human cancers were identified based on computational analysis of data in COSMIC [15].

The RAS genes, *KRAS*, *HRAS*, and *NRAS*, are the most frequently mutated proto-oncogenes in human cancers in the United States and are responsible for 43% of all cancer deaths. Among the RAS genes mutations in *KRAS* are the most abundant and are associated with poorer clinical outcomes [16]. While missense mutations at G12, G13, and Q61 in *KRAS* are canonical drivers of lung, pancreas, and colorectal cancers [16], overexpression of wild-type KRAS has been observed in head and neck [17], endometrial [18], ovarian [19], testicular [20], lung [21], gastric [22], colon [23], and bladder cancers [24]. Endometrial cancer patients whose tumors overexpress wild-type (WT) KRAS have a lower probability of survival [18]. Individuals having colon cancers that overexpress WT KRAS are resistant to EGFR monoclonal antibody therapies [23]. The codons found in the *KRAS* gene, in contrast to those in *HRAS*, have been selected for low protein expression, and it has been hypothesized that *KRAS* cancers may be more common because low expression of mutant KRAS protein promotes hyperplasia but not senescence [25–27], allowing additional mutations to be accumulated on the path to cancer.

Here we describe experiments showing that all synonymous codon replacements at *KRAS* G12, G13, and G60 substantially increase KRAS protein expression in stably transfected NIH3T3 cells. Further, the phenotypes of many of these cell lines are significantly altered toward more transformed states. Because these synonymous mutations in *KRAS* have been found in human cancers, we suggest that testing for the mutational status of *KRAS* in cancer patients should not systematically exclude synonymous codon replacements.

## Results

### Single synonymous mutations in KRAS cause increases in KRAS protein expression

**Fig 1:**
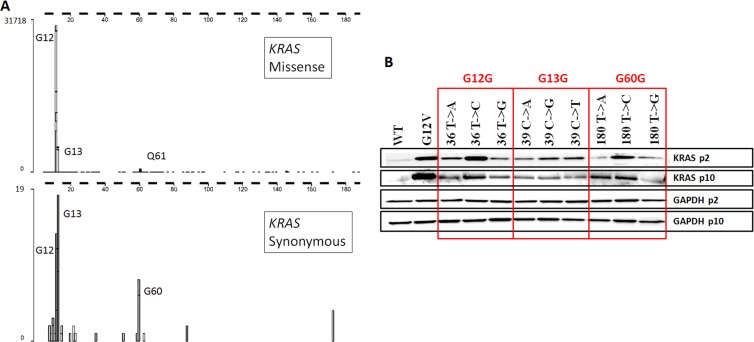
Synonymous KRAS mutations found in human tumors increased KRAS protein expression. (A) Positions and frequencies of missense (top panel) and synonymous (bottom panel) mutations found in *KRAS* genes in human cancers, as of 27 January 2016. Source: Catalogue of Somatic Mutations in Cancer. (B) NIH_3_T_3_ cell lines stably transfected with *KRAS* containing single synonymous glycine mutations at G12, G13, or G60 overexpress KRAS protein compared to wild type *KRAS*. p2 = passage 2, p10 = passage 10.

The classic sites of missense mutations in *KRAS* genes found in human cancers are at G12, G13, and Q61. Intriguingly, the most frequent synonymous mutations in *KRAS* found in human tumors are at almost the same locations, G12, G13, and G60 (http://cancer.sanger.ac.uk/cosmic; [28] (Fig 1A). These mutations have not been identified as SNPs in the healthy population [29].

To investigate whether these synonymous glycine mutations contributed to changes in KRAS protein expression, we constructed plasmids encoding the wild type KRAS amino acid and nucleotide sequence, the missense G12V oncogenic mutant, and nine different single-nucleotide changes encoding synonymous glycine codons at G12, G13, or G60 (using primers in S1 Table), and we used these plasmids to establish stable NIH3T3 cells lines. Based on inspection of the adherent cultures during drug selection, each of the eleven stable cell lines comprised between approximately 10 and 30 independent clones (data not shown). At the end of the selection period, each cell line was cultured as a pooled population.

Lysates were prepared from each cell line after 2 and 10 passages and analyzed by immunoblots for KRAS protein expression (Fig 1B). Strikingly, all the KRAS synonymous mutation cell lines expressed much more KRAS protein, between 2 and 14 fold (Table 1), at early and late passage, compared to cells stably expressing the WT *KRAS* nucleotide sequence. Increases in KRAS protein expression trended with human and mouse glycine codon usage at G12 and G60, but not at G13 (Table 1), possibly because of a highly conserved CpG motif spanning the G13V14 codon pair. There were no known miRNA binding sites [30] that encompassed any of the synonymous changes, and these codons were not near splice sites. Secondary structures of mRNAs and predicted free energies [31] varied slightly, but all synonymous mutant *KRAS* mRNA levels were within two-fold of the WT sequence based on droplet digital PCR results of transiently transfected cells (S1 Fig). We investigated the activation states of MAPK and PI3K signaling pathways in the synonymous mutant cell lines, and while we observed reproducible differences between the cell lines, no pattern emerged associating either MAPK signaling or PI3K signaling with levels of KRAS protein expression (S2 Fig). This is consistent with previous reports which failed to correlate signaling patterns with different *missense* mutations [32–34].

**Table 1:** Glycine codon usage and KRAS protein expression in pooled stable cell lines.

Glycine Codon Usage	G12 Codon	Relative KRAS protein expression*	G13 Codon	Relative KRAS protein expression*	G60 Codon	Relative KRAS protein expression*
Least oftenMost often	GGT (wt)	1.0	GGT	6.1	GGT (wt)	1.0
	GGG	3.2	GGG	5.8	GGG	2.4
	GGA	5.4	GGA	3.4	GGA	4.0
	GGC	14.3	GGC (wt)	1.0	GGC	13.4

*Average of passage 2 and passage 10 KRAS values as determined by ImageJ densitometry.

### Single synonymous mutations cause increases in proliferation and saturation density

KRAS effector signaling drives proliferation [16], and increased proliferative status is associated with cellular transformation [35]. We determined doubling times for all the cell lines each day for 7 days to determine if the synonymous mutation-mediated increases in KRAS protein expression correlated with proliferation rates and final cell densities (Table 2). All the cells reached confluence by day 7, many of them by day 5, and doubling rates were calculated each day during the experiment. Cells expressing WT KRAS grew the slowest (minimum observed doubling time of 47 hours). Proliferation rates paralleled KRAS protein expression for nine of the eleven lines (more KRAS protein, faster growth). Increased saturation density is a characteristic of transformed cells [35], and all the cell lines expressing the synonymous mutants reached final maximum cell densities more than 2-fold greater than those expressing the WT gene during the 7-day assay.

**Table 2:** Single synonymous mutations changed KRAS expression, doubling times, and cell densities.

	Minimum Doubling Time (hr)	KRAS Protein Expression*	Maximum Cells (x 10^5^)
WT	**44.0**	1.0	7.3
G12V	**20.5**	16.6	51.9
36 T->A	**28.7**	5.4	15.9
36 T->C	**22.5**	14.3	18.7
36 T->G	**34.2**	3.2	18.8
39 C->A	**30.0**	3.4	16.3
39 C->G	**28.5**	5.8	16.1
39 C->T	**27.1**	6.1	16.8
180 T->A	**25.1**	4.0	18.1
180 T->C	**24.6**	13.0	19.2
180 T->G	**28.4**	2.4	21.4

*Normalized to WT = 1.0

### Some synonymous mutant cell lines lose contact inhibition and exhibit a MAPK-dependent refractile appearance

**Fig 2:**
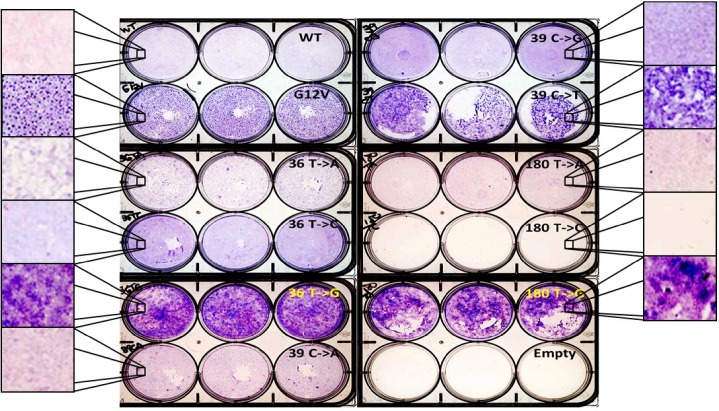
Some silent mutant cell lines exhibited loss of contact inhibition. Cells were fixed and stained after 21 days of growth in triplicate wells. Enlarged areas are representative images. The 180 T->C cells formed a monolayer and detached within the first week of culture and were lost during fixation and staining (S1 Movie and S3 Fig).

The RAS genes were originally identified as oncogenes based on their ability to cause NIH3T3 cells to lose contact inhibition and form colonies / foci [36]. To investigate whether the synonymous mutant cell lines exhibited a similar transformed phenotype, we performed colony forming assays (Fig 2 and S3 Fig). While WT cells were contact-inhibited during the 21-day assay, all the synonymous mutant cell lines displayed altered behaviors, ranging from slight increases in crystal violet staining (36 T->C, 180 T->A, 180 T->C) to densely stained colonies (36 T->G, 39 C->T, and 180 T->G). Surprisingly, all three of the cell lines in this latter group only moderately overexpressed KRAS protein (2–6 fold over WT; Fig 1B). In addition, in two experiments all replicates of one line, 180T->C, detached as a cell monolayer from the polylysine-coated plastic as the cells reached confluence (S1 Movie shows the release in one well over two minutes). The cells remained floating as a sheet with no visible debris for the duration of the experiment (data not shown).

**Fig 3:**
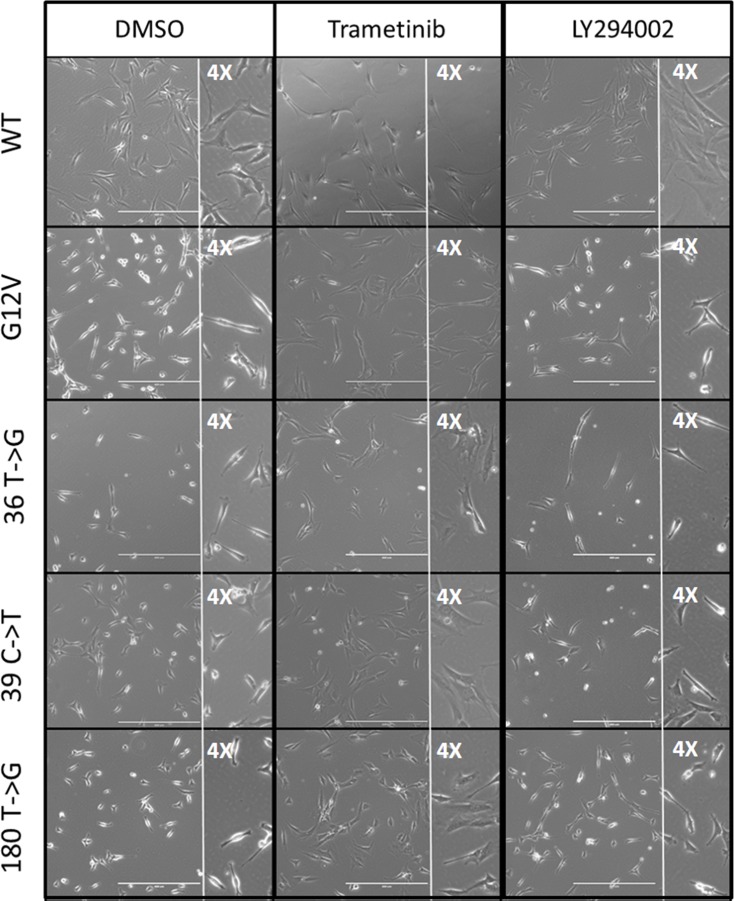
Refractile morphology of silent mutant cell lines was MAPK, but not PI3K, dependent. Cells were plated at low density and photographed with phase contrast microscopy before or after 24 hours of treatment. Left column: vehicle only. Center column: Mek 1/2 inhibitor (1 μM trametinib). Right column: PI3K inhibitor (5 μM LY294002). Insets are 4X views.

A refractile and more rounded morphology is characteristic of cellular transformation [35] and has been associated with RAS-driven transformation [37]. The three cell lines that showed reduced contact inhibition in the colony forming assay (36 T->G, 39 C->T, and 180 T->G) were also more refractile and more rounded than WT cells under phase contrast microscopy. Treatment with the MEK inhibitor trametinib (1 μM) for 24 hours reverted the refractile and rounded morphology in all three lines nearly to the appearance of the WT DMSO-treated cells (Fig 3), and the response was dose-dependent. In contrast, after treatment with the PI3K inhibitor LY294002 (5 μM), the refractile character of the three cell lines remained (S4 Fig). Thus the refractile appearance of these synonymous mutant cells lines was dependent upon MAPK, but not PI3K, signaling.

### Synonymous mutant cell lines and spheroids are more invasive than the WT cell line

**Fig 4:**
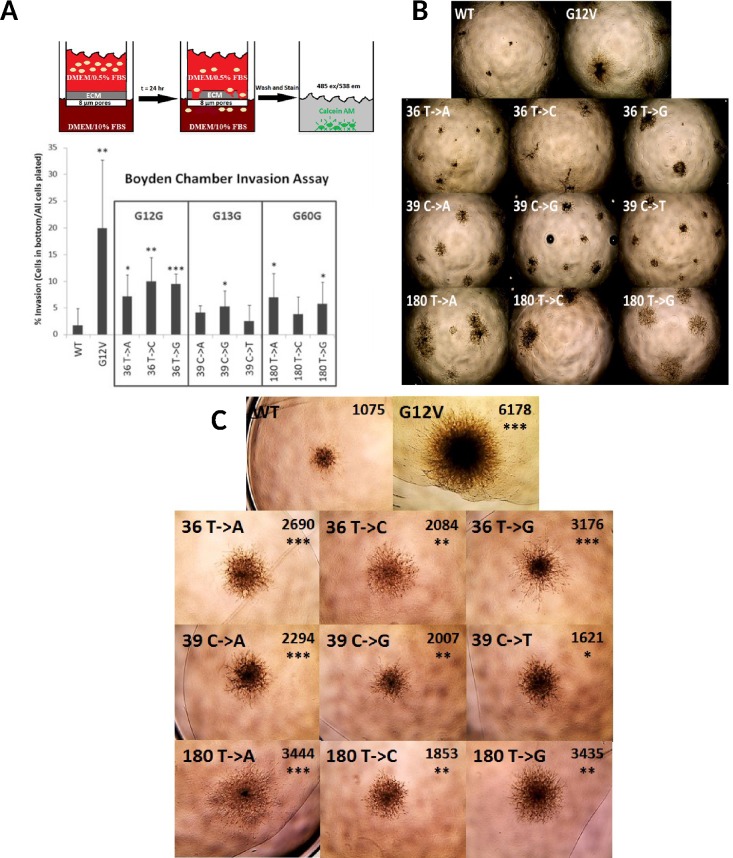
All silent mutant cell lines were more invasive than the WT cell line. (A) Top Panel: Diagram depicting the Boyden chamber assay. Cells were deprived of serum and allowed to invade through the extracellular matrix toward the chemo-attractant (FBS) for 24 hours before staining with Calcein-AM. Bottom Panel: Percent invasion of the cell lines: ratio of cells that invaded through the ECM to the total number of cells initially plated in the top chamber. (B) Growth in ECM-coated wells. 56X total magnification. (C) Growth of spheroids under ECM. 56X total magnification. Average surface areas (pixels) and statistical significance for each set of spheroids (N = 3) are shown.

Invasion is a requirement for metastasis in cancer cells [35]. We evaluated invasion of the WT, G12V, and synonymous mutant cell lines using three standard assays. First the lines were grown in a Boyden chamber in which a layer of extracellular matrix (ECM) was coated on top of a membrane with 8 μm pores in the upper chamber. Every synonymous mutant cell line showed increased invasion through the ECM toward the chemoattractant compared to the WT cell line (Fig 4A). Next we cultured each cell line in wells precoated with extracellular matrix (ECM; "top assay"), in which the cells had to degrade the matrix in order to proliferate and survive (Fig 4B). The WT cells formed small, compact masses, while the G12V cells grew into much larger cell aggregates with abundant invadopodia into the matrix. All the synonymous mutant cell lines grew into larger colonies with more protrusions than WT cells. Finally, we formed spheroids with each cell line and then overlaid them with ECM, so that growth required invasion into the matrix (Fig 4C). The growth of the synonymous mutation spheroids was less than that of the G12V line but always significantly greater than the WT spheroids. These assays showed that stable NIH3T3 cell lines expressing *KRAS*-4B with single synonymous substitutions at G12, G13, and G60 were always more invasive than cells expressing the WT protein.

We aggregated the above data by ranking each cell line for its similarity to the negative control wild-type cell line and the positive control G12V cell line in each of 7 assays (Table 3), and used the 4 phenotypic assays to construct a "transformation index". By this measure, all the synonymous mutation cell lines were more transformed than the WT cell line and less transformed than the G12V cell line.

**Table 3:** Relative scores of cell lines for KRAS expression, signaling by phospho-Erk1/2 and phospho-Akt, and transformation phenotypes.

	KRAS Expression and Signaling			Transformation Assays				

	KRAS Expression	pErk 1/2	pAkt	Prolifera-tion	Maximum Cells	Loss of Contact Inhibition	Invasion	Transformation Index*
WT	+	++	++	+	+	+	+	4
G12V	++++	+++	++++	++++	++++	++++	++++	16
36 T->A	++	++	+	++	++	++	++	8
36 T->C	+++	++	+	++++	+++	+	+++	11
36 T->G	++	++	++	++	++	+++	+++	10
39 C->A	++	++	+	++	++	++	++	8
39 C->G	++	+	+	+++	++	+	++	8
39 C->T	++	+	+	++	+++	+++	++	10
180 T->A	++	++	+	+++	++	+	++	8
180 T->C	+++	+++	++	+++	++	+	++	8
180 T->G	++	++++	++	+++	+++	+++	++	11

* total of transformation scores (+ = 1, ++ = 2, +++ = 3, ++++ = 4)

## Discussion

Historically, synonymous mutations in cancer genes have been discounted as unimportant (for example [38]). It is indeed probable that they represent a minority of the genome changes that contribute to cancer. However, accumulating data from sequencing the exomes and genomes of human cancers have revealed interesting hotspots for synonymous changes, especially considering that many such mutations do not find their way into publications. In fact, the G12, G13, and G60 synonymous mutations catalogued in COSMIC show up >6 times more often from whole exome sequencing than from literature curation, and some research in which synonymous mutations were found at these locations is not catalogued in COSMIC [39].

Here we have explored the consequences of synonymous mutations that cluster in *KRAS*. Importantly, there are clusters of synonymous mutations in other classic cancer genes in the COSMIC database (http://cancer.sanger.ac.uk/cosmic; [28], including *KIT*, *NOTCH1*, *BRCA1*, and *CTNNB1* (ß-catenin)). Especially notable in the case of *KRAS* is the near coincidence of the clusters of synonymous mutations at G12, G13, and G60, with the classic missense mutations at G12, G13, and Q61 that drive human cancers (Fig 1A). This clustering of synonymous mutations near sites of missense mutations has been observed for synonymous mutations that drive human cancers [4]. We considered the possibility that the clusters arose from ascertainment bias. Examination of the publications behind the data showed that the sequences were derived from both Sanger and NextGen methods, and that the Sanger data usually encompassed entire exons (S2 Table). These considerations led us to ask whether the clusters of synonymous mutations might have biological significance.

Our biochemical and phenotypic experiments revealed substantial changes associated with the synonymous mutations. All the stable NIH3T3 cell lines expressing any of the transfected synonymous, single-nucleotide altered *KRAS* genes expressed substantially (2–14 fold) more KRAS protein than did the cells expressing the control wild-type gene (Fig 1B). Since each of the cell lines (wild type, G12V, and synonymous) comprised 10–30 independent clones, we judged explanations based on dominance of rare clones to be unlikely. Levels of KRAS expression paralleled glycine codon usage at G12 and G60 but not G13 (Table 1). Interestingly, the wild-type G13 codon is part of the rarest GV codon pair in humans, GGC GTA, and that codon pair is in fact the 21st rarest of all 3721 human codon pairs [9]. Thus it is reasonable to hypothesize that the increases in KRAS expression we saw were due to relief from rare codon (G12 and G60) or rare codon pair (G13) usage.

We conducted an extensive examination of MAPK and PI3K signaling pathways in the cell lines without finding any correlations that prevailed across all the synonymous mutants (S2 Fig). We are not aware of a similar analysis across a large number of KRAS missense mutants in 3T3 cells, but it is well known that perturbations in RAS pathway signaling engage feedback mechanisms that limit the duration of changes in, for example, phospho-Erk [32–34]. In agreement with others [35, 40], we think it is likely that transformed phenotypes are the consequence of the integrated output of multiple signaling pathways. For example, it is plausible that small (2 fold), medium (5 fold) and large (>10 fold) increases in the amounts of wild-type KRAS protein would trigger different downstream signaling and compensatory feedback responses.

We also suggest that the high conservation of codon usage at G12, G13, and G60 of KRAS genes (S3 Table) compared to HRAS may be connected to the biochemical and cell biology phenotypes we report here. First, KRAS is the most potent of the gain-of-function oncogenes, since it drives more human cancers than any other [16]. Evolution may have selected for inefficient translation of KRAS mRNA, as it does in other eukaryotes [6,7], since both synonymous (reported here, and see [27]) and oncogenic missense (our unpublished data) mutations dramatically increase the amounts of KRAS protein in transfected cells. In the context of a tissue in a live animal, single synonymous mutations could cause large increases in MAPK signaling that could push cells into apoptosis or senescence, and only rarely initiate a process leading to cancer [26]. Second, we favor codon usage and increased expression of wild-type protein to explain the results we see for synonymous mutations at G12 and G13. However, mutations at G60 could potentially change the folding of the protein itself. Slow translation at G60 might be necessary to allow the switch 1 region of KRAS protein, having left the exit tunnel of the ribosome, to fold properly before the rest of the protein is synthesized. Reducing the pause caused by slow translation at G60 might result is a population of KRAS molecules with differential GAP, GEF, and effector binding and thus altered signaling properties.

**Fig 5:**
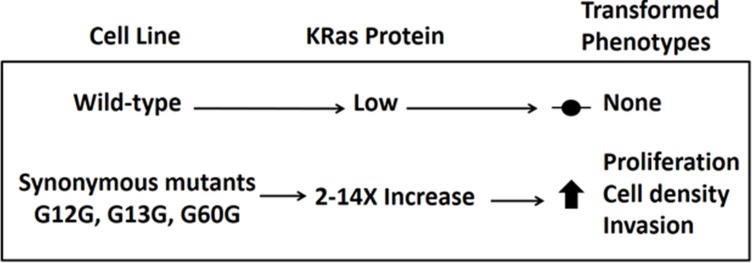
Working model. The WT *KRAS* nucleotide sequence has been evolutionarily conserved to minimize protein expression, especially at G12, G13, and G60. Any synonymous mutation at those positions leads to overexpression of KRAS protein and subsequent increases in proliferation, maximum cell density, and invasion in NIH_3_T_3_ cells.

We found that the MEK inhibitor trametinib reverted the refractile morphology of the colony-forming synonymous mutant cell lines toward a wild-type appearance (Fig 3). In addition, we found an association between synonymous-mutant driven levels of KRAS protein overexpression and transformed phenotypes. All synonymous mutant cell lines divided more rapidly, and grew to higher densities in culture (Table 2). Finally, in three independent assays all the synonymous mutant lines invaded extracellular matrix more aggressively than the wild-type cells (Fig 4). Our observations on the effects of synonymous mutations in KRAS-4B are summarized in Table 3 and Fig 5.

It has been previously observed that expression of mutant KRAS protein is restrained by codon usage compared to HRAS [25–27]. In those reports, increasing extents of *KRAS* codons were substituted with *HRAS* codons, with the result that progressively more KRAS protein was expressed, and cells expressing higher amounts of KRAS formed smaller tumors in mice. In contrast, our observations are based on single nucleotide changes in the wild-type coding sequence, and resulted in KRAS protein levels higher than WT but lower than G12V KRAS. The single nucleotide changes we investigated have been found in human tumors in the absence of the classic *KRAS* missense mutations (http://cancer.sanger.ac.uk/cosmic; [28]).

It is remarkable that these small changes had such large effects on levels of KRAS protein. We note that the codons used for these 3 glycines in humans are highly conserved in KRAS homologs in 57 vertebrate species (92% conserved) compared to HRAS (48% conserved) (S3 Table). Thus our observations, combined with the clustered occurrence of the same mutations in human cancers and the high degree of conservation of these codons in vertebrates, indicate that maintenance of these particular glycine codons is important for maintaining low levels of KRAS protein in normal cells.

Chromosomal amplifications and translocations are frequent drivers of overexpression that lead to cancer [20]. Our observations, combined with previously published reports [4, 15], data such as those in COSMIC, and intriguing individual reports [39, 41], suggest that synonymous mutations in KRAS, and possibly other cancer genes should also be regarded as potential drivers of overexpression and participants in tumorigenesis / transformation. Because mutant *KRAS* genes are major drivers of common cancers, standardized tests for mutations in *KRAS* [42] are being developed and used in trials of tailored cancer therapies ("precision medicine"). Unfortunately, all such tests at present are designed to be blind to synonymous mutations in *KRAS*. We suggest that "silent" changes in *KRAS*, and perhaps other important cancer genes, should be incorporated into decisions about the most appropriate therapies for human patients.

## Materials and Methods

### Plasmids

Synonymous mutations at G12, G13, and G60 (nucleotide positions 36, 39, and 180, respectively) were introduced into an entry clone containing the human WT *KRAS*-4B coding nucleotide sequence using the Q5^®^ Site-Directed Mutagenesis Kit (New England Biolabs, Inc., Ipswich, MA) according to the manufacturer’s instructions with the primers listed in S1 Table. Entry clones were validated by Sanger sequencing in the forward and reverse directions. WT, G12V, and synonymous mutant expression clones were generated using Gateway cloning [43] into a destination vector, pDest 720 (S5 Fig) with a CMV promoter to drive expression and an SV40 promoter to drive zeocin resistance gene for selecting stables.

### Cell Culture

Mouse embryonic fibroblast NIH3T3 cells were purchased from the American Type Culture Collection (ATCC, Manassas, VA). NIH3T3 cells were maintained and propagated at 37°C, 5% CO_2_, in Dulbecco’s Modified Eagle Medium (DMEM) (ATCC, Manassas, VA) supplemented with 10% fetal bovine serum (FBS) (Hyclone, Logan, UT). All experiments were begun when cells were between passage 2 and passage 5, and cell viabilities were maintained at ≥90%. Serum-starvation conditions were generated by culturing cells for 18–24 hours in 20-fold reduced serum concentrations (DMEM + 0.5% FBS). NIH3T3 cell lines were validated with the mouse cell line authentication STR profile as previously described [44]. Cell lines tested negative for mycoplasma throughout the course of the experiments.

To make stable cell lines, cells were transfected with FuGENE HD (Promega Life Sciences, Madison, WI) in Optimem (Life Technologies Corp, Carlsbad, CA) with a reagent to DNA ratio of 2.5:1 (v/v) at 6.25 pg plasmid DNA/cell. After 48 hours, 2 x 10^5^ viable cells were plated in tissue culture treated six-well plates and propagated in DMEM + 10% FBS + 700 μg/ml zeocin (Life Technologies Corp., Carlsbad, CA), for three weeks. Fresh zeocin-containing DMEM + 10% FBS was exchanged for the spent culture medium every three to four days. Stably-selected cells were grown as pools and maintained in DMEM + 10% FBS with 350 μg/ml zeocin.

### Immunoblotting

Immunoblots were performed by loading 20 μg of total protein (as determined by BCA assay (Pierce Biotechnologies, Waltham, MA) using BSA as the standard) from a cell lysate per gel lane. Lysis buffer composition was 150 mM NaCl, 20 mM Tris pH 7.5, 1% Triton X-100, 1 mM EDTA, including cOmplete™, EDTA-Free, Protease Inhibitor Cocktail Tablets (1 tablet per 10 ml lysis buffer) (Sigma Aldrich, St. Louis, MO) and PhosSTOP™ phosphatase inhibitors (1 tablet per 10 ml lysis buffer) (Sigma Aldrich, St. Louis, MO). Gels were transferred to nitrocellulose membranes using the semi-dry iBlot transfer system (Life Technologies Corp, Carlsbad, CA) for 7 minutes. All primary (1:1000) and secondary (1:2000) antibody incubations were in 3% dry milk (BioRad, Hercules, CA). Membranes were developed after a 2-minute incubation with SuperSignal™ West Femto Maximum Sensitivity Substrate (Life Technologies Corp, Carlsbad, CA). Anti-KRAS (cat # WH0003845M1) was purchased from Sigma Aldrich (St. Louis, MO). Anti-Mek1/2 (cat # 9122), anti-phospho-Mek1/2 Ser 217/221 (cat # 9121), anti-Erk1/2 (cat # 9102), anti phospho-Erk1/2 Thr202/Tyr204 (cat # 9101, and E10 cat# 9106), anti-Akt (cat # 9272), anti-phospho-Akt Ser473 (cat # 9271), goat anti-rabbit IgG HRP-linked secondary antibody (cat #7074), and horse anti-mouse IgG HRP-linked secondary antibody (cat # 7076) were purchased from Cell Signaling Technologies (Danvers, MA). Anti-GAPDH (cat #2275-PC-100) was purchased from Trevigen, Inc. (Gaithersburg, MD). Band intensities were quantified using ImageJ software (National Institutes of Health, Bethesda, MD). Active (GTP-bound) KRAS (Pierce Biotechnologies, Waltham, MA) pulldowns were performed according to the manufacturer’s instructions.

### Transformation Assays

#### Growth Curves

1.25 x 10^5^ viable cells for each cell line were plated into 7 separate 25 cm^2^ tissue culture-treated flasks (Corning Cat #430639). To determine the total cell number and viability, one flask for each construct was harvested each day and cells were counted in duplicate in a TC-20 cell counter (BioRad) in 0.2% Trypan Blue. The doubling time was recorded as the fastest doubling time between days 2 and 5 for each construct in the assay. These assays were performed in duplicate, and the peak doubling times and saturation densities were averaged for the two experiments.

#### Loss of Contact Inhibition

5 x 10^4^ viable cells for each cell line were seeded in triplicate into 6-well tissue culture treated plates (Costar Cat #3506). Cells were grown for 21 days at 37°C, 5% CO_2_, in a humid environment. On day 21, culture medium was removed, and cells were fixed (3:1 ratio of methanol to glacial acetic acid) and stained with 0.1% crystal violet.

#### Boyden Chamber Invasion Assays

After a 20-hour serum-starvation, cells were harvested, counted, and 7 x 10^3^ cells were added to the top of a Boyden chamber containing 8 μm pores (Trevigen 3455-096-01) overlaid with extracellular matrix (Trevigen 3455-096-K). The cells were allowed to invade through the extracellular matrix and pores toward a chemo-attractant (DMEM + 10% FBS) or culture medium (DMEM) for 24 hours. Cells were washed, rinsed from the bottom of the chamber, and incubated with Calcein-AM (Trevigen, Inc., Gaithersburg, MD). Fluorescence was measured in a Molecular Devices SpectraMax M5 plate reader with 485 nm excitation, 538 nm emission, and a 530 nm cutoff filter. Percent invasion was calculated by subtracting the average number of cells which invaded in the absence of a chemo-attractant (DMEM) from the average number of cells which invaded toward a chemo-attractant (average of sextuplicates) and dividing by the number of viable cells plated per well.

#### 3D Cell Culture Invasion “Top Assay”

Equal numbers of cells for each cell line (3 x 10^4^ cells) were plated in duplicate wells of a 3D Culture Matrix™ BME Coated 96 Well Plate (Trevigen, Inc. Gaithersburg, MD). Cellular self-organization on the extracellular matrix was monitored and imaged microscopically over a period of 12 days.

### 3D Spheroid Invasion “Embedded” Assay

Cell lines were harvested and resuspended in DMEM + 10% FBS + 1X Spheroid Formation ECM (Trevigen, Inc., Cat No. 3500-096-01, Gaithersburg, MD). Equal numbers of cells for each cell line (3 x 10^3^ viable cells per 50 μl DMEM + 10% FBS + 1X Spheroid Formation ECM) were plated in triplicate for each construct into a 3D Culture Qualified 96 Well Spheroid Formation Plate (Trevigen, Inc., Cat No. 3500-096-K, Gaithersburg, MD) and centrifuged at 200 x *g* in a swinging bucket rotor (Model 5810R) for 3 minutes. Cells were incubated at 37°C, 5% CO_2_ for 72 hours to promote spheroid formation (one spheroid per well). After 72 hours, the 96-well plate was cooled at 4°C for 15 minutes before adding 50 μl of Invasion Matrix (Trevigen, Inc., Cat. No. 3500-096-03, Gaithersburg, MD). The 96-well plate was then transferred to a 37°C incubator for 1 hour to promote gel formation before 100 μl of DMEM + 10% FBS were overlaid. Cells were incubated at 37°C, 5% CO_2_ for 7 days and wells were microscopically imaged each day. Images were analyzed with ImageJ software (National Institutes of Health, Bethesda, MD, http://imagej.nih.gov/ij/) for total surface area (in pixels according to the manufacturer's instructions) to evaluate 3D cell culture invasion.

### Drug Treatments and Refractility

5 x 10^3^ cells from the three cell lines with loss of contact inhibition and the WT and G12V lines were plated into six well tissue-culture treated plates. After 24 hours, medium was removed and replaced with medium containing 1% DMSO (Sigma Aldrich, St. Louis, MO), 1% DMSO containing trametinib (final concentrations 10 pM to 1 μM; GlaxoSmithKline, Brentford, UK), or 1% DMSO containing LY294002 (final concentrations 500 pM to 50 μM; Eli Lilly, Indianapolis, IN). After 24 hours of drug treatment, cells were imaged using an EVOS FL microscope in phase-contrast mode.

### miRNA

Predictions of miRNA binding sites in the coding region of the *KRAS* gene were generated with the SVM-based software tool, MiRPara [30].

### Statistical Analysis

Statistical analyses for immunoblotting, Boyden chamber spheroid assays, and spheroid invasion assays were performed in Microsoft Excel using the Student's *t* test assuming equal variances. Data were presented as mean values ± standard deviation. Confidence intervals were denoted with one asterisk (*p* values <0.05), two asterisks (*p* values <0.01) or three asterisks (*p* values <0.001) in the appropriate figures.

## Supporting Information

S1 Fig Predicted secondary structure and free energy of synonymous mutant KRAS mRNAs. (A) mRNA levels from droplet-digital PCR varied by less than two-fold among cells transiently transfected with synonymous mutant constructs. (B) Global free energy and predicted secondary structure of synonymous mutant mRNAs. (C) Local free energy and predicted secondary structure of synonymous mutant mRNAs.
(TIF)

S2 Fig Synonymous mutant cell lines exhibit altered PI3K and MAPK signaling pathways. Synonymous mutant cell lines all have similar amounts of Akt, Mek, and Erk proteins, but have altered phosphorylation states, as measured by pAkt (PI3K pathway), pMek (MAPK pathway), and pErk (MAPK pathway) compared to wild-type cells, indicating changes to activation status. PI3K and MAPK signaling activation is similar at passage 2 (p02) and passage 10 (p10) as measured by pAkt and pErk.
(TIFF)

S3 Fig Microscopic images from the colony forming assay. All the cell lines had distinct microscopic morphologies during growth to and past confluence. The scale bars are 1000 microns.
(TIFF)

S4 Fig Refractile appearance of cell lines was reversed with a MEK inhibitor in a dose-dependent manner. Synonymous mutant cell lines that have lost contact inhibition have a rounded, refractile morphology. With increasing concentrations of trametinib, the cells revert toward a flattened, spread out morphology characteristic of the wild-type cell line.
(TIF)

S5 Fig Plasmid Construction for Cell Lines. Gateway cloning was employed to replace the *ccdB* and *CAT* genes in pDest720 with a KRAS gene. Eleven different KRAS plasmids (WT, G12V, 9 silent mutants) were generated, each containing a unique KRAS gene and a zeocin resistance gene.
(TIFF)

S1 File mRNA methods. Methods include mRNA quantification and structure prediction.
(DOCX)

S1 Movie Time lapse of a sheet of 180 T->C cells detaching from the culture vessel surface. Frames were taken every 10 seconds for 2 minutes.
(AVI)

S1 Table Primers used to introduce synonymous mutations in KRAS. (TIF)

S2 Table Synonymous mutations in KRAS from COSMIC references. (TIF)

S3 Table Conservation of third-position bases in glycine codons G12, G13, and G60 in *KRAS* and *HRAS*. DNA sequence logos were constructed according to [45].
(TIFF)
