# Supplementary figures and images for: Single Synonymous Mutations in KRAS Cause Transformed Phenotypes in NIH3T3 Cells
Source: PLoS One. 2016 Sep 29;11(9):e0163272. doi: 10.1371/journal.pone.0163272 (PMC5042562; doi:10.1371/journal.pone.0163272)

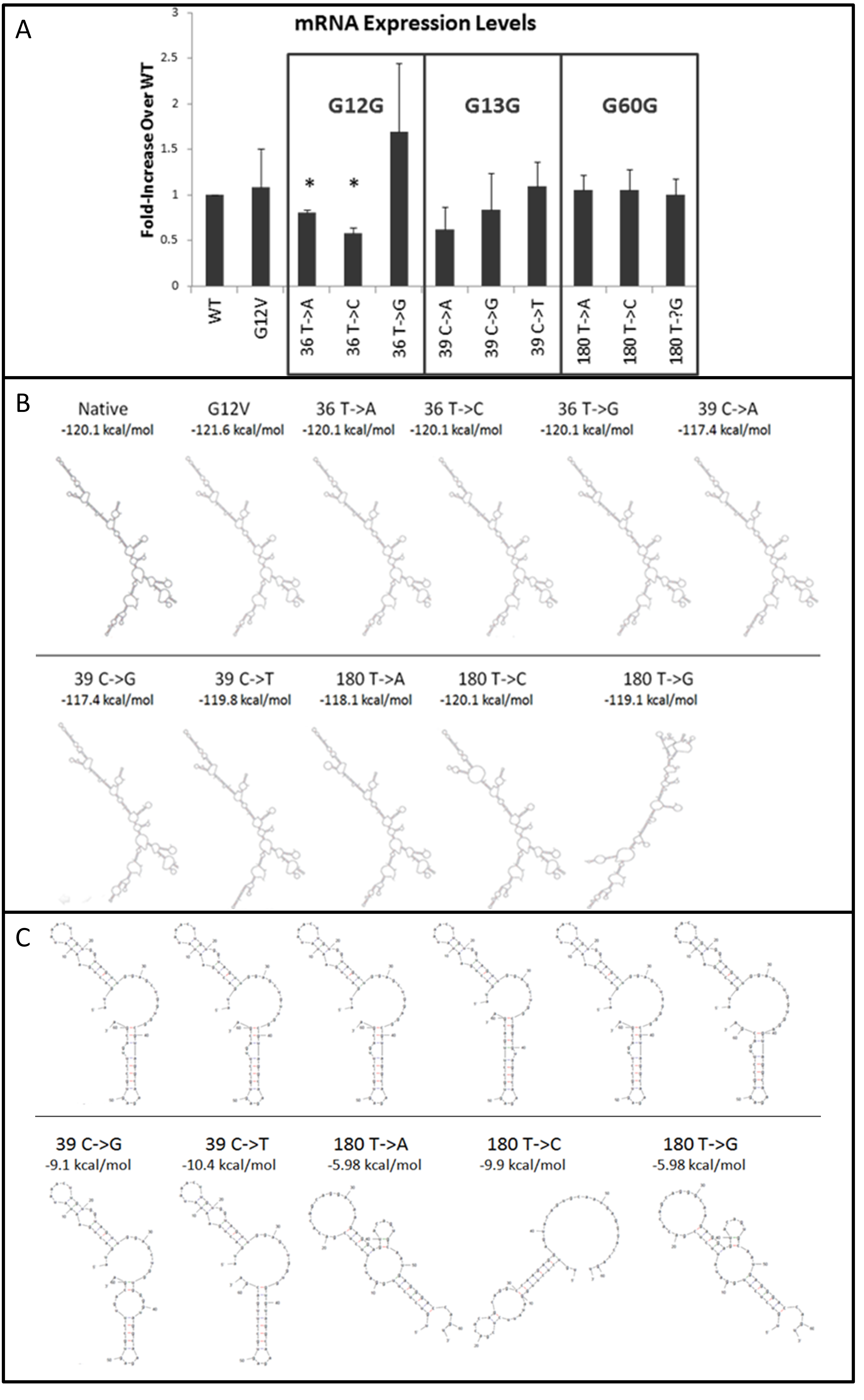

Supplement: S1 Fig — (A) mRNA levels from droplet-digital PCR varied by less than two-fold among cells transiently transfected with synonymous mutant constructs. (B) Global free energy and predicted secondary structure of synonymous mutant mRNAs. (C) Local free energy and predicted secondary structure of synonymous mutant mRNAs. (TIF) [file pone.0163272.s001.tif]

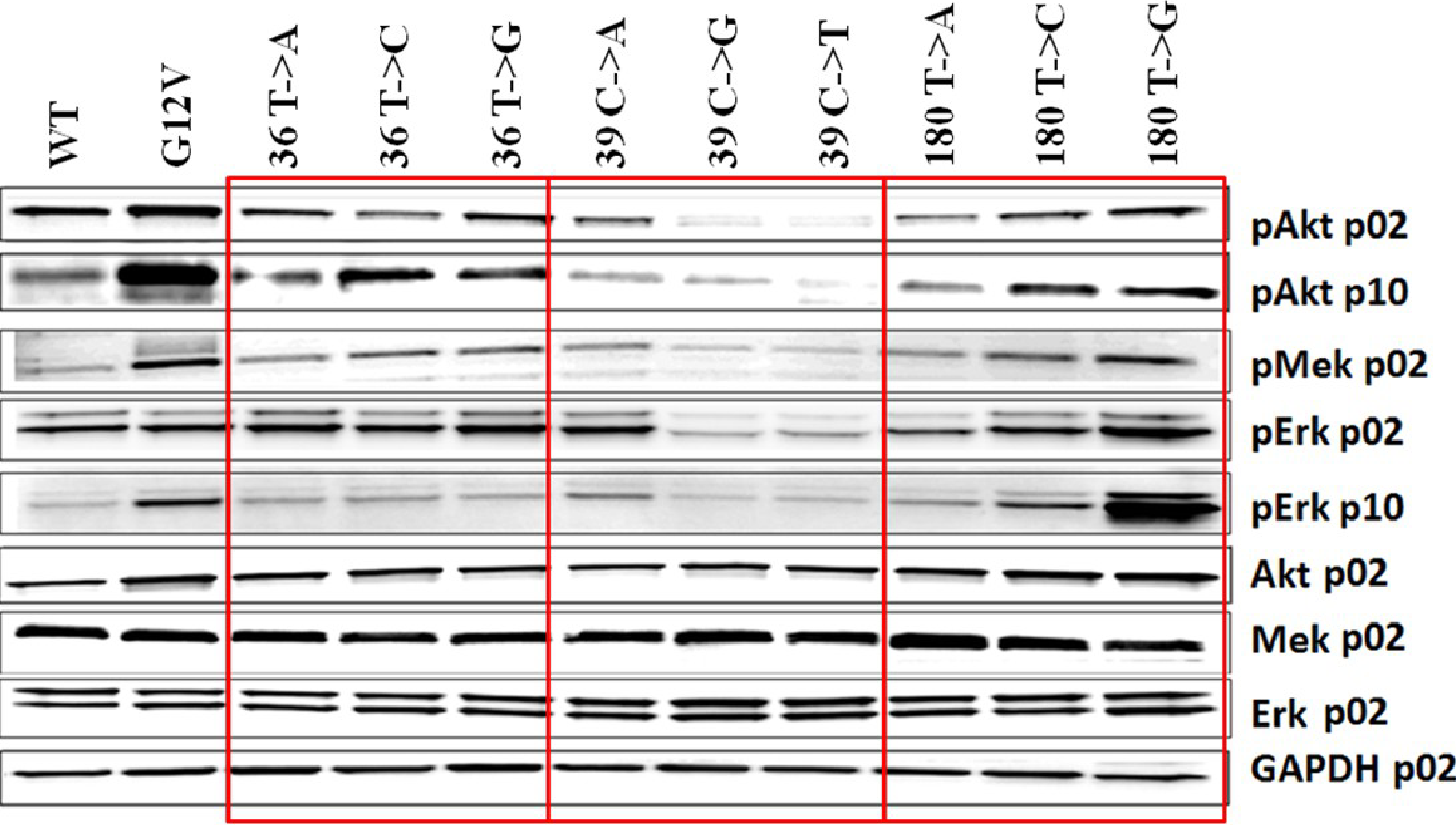

Supplement: S2 Fig — Synonymous mutant cell lines all have similar amounts of Akt, Mek, and Erk proteins, but have altered phosphorylation states, as measured by pAkt (PI3K pathway), pMek (MAPK pathway), and pErk (MAPK pathway) compared to wild-type cells, indicating changes to activation status. PI3K and MAPK signaling activation is similar at passage 2 (p02) and passage 10 (p10) as measured by pAkt and pErk. (TIFF) [file pone.0163272.s002.tiff]

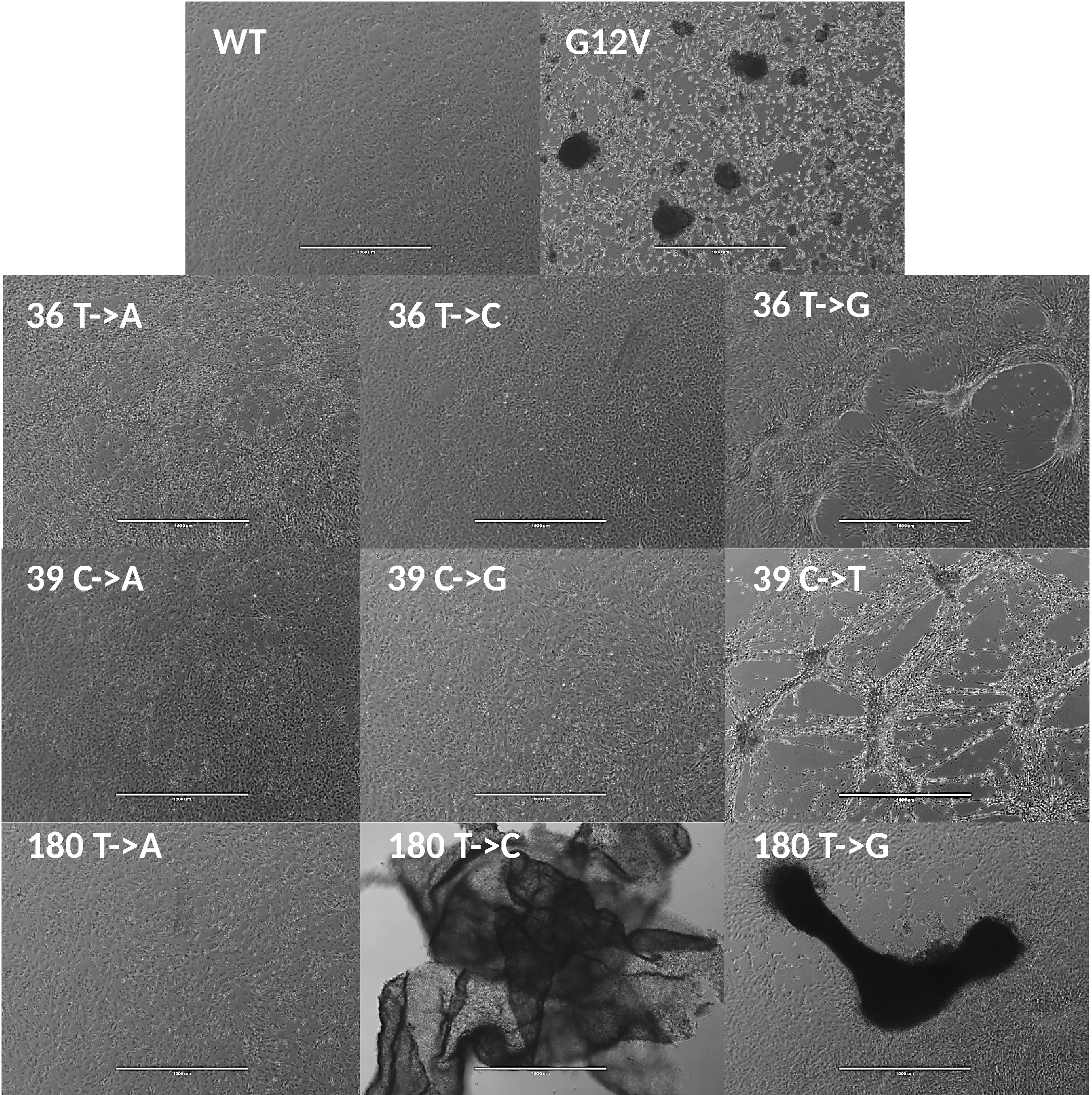

Supplement: S3 Fig — All the cell lines had distinct microscopic morphologies during growth to and past confluence. The scale bars are 1000 microns. (TIFF) [file pone.0163272.s003.tiff]

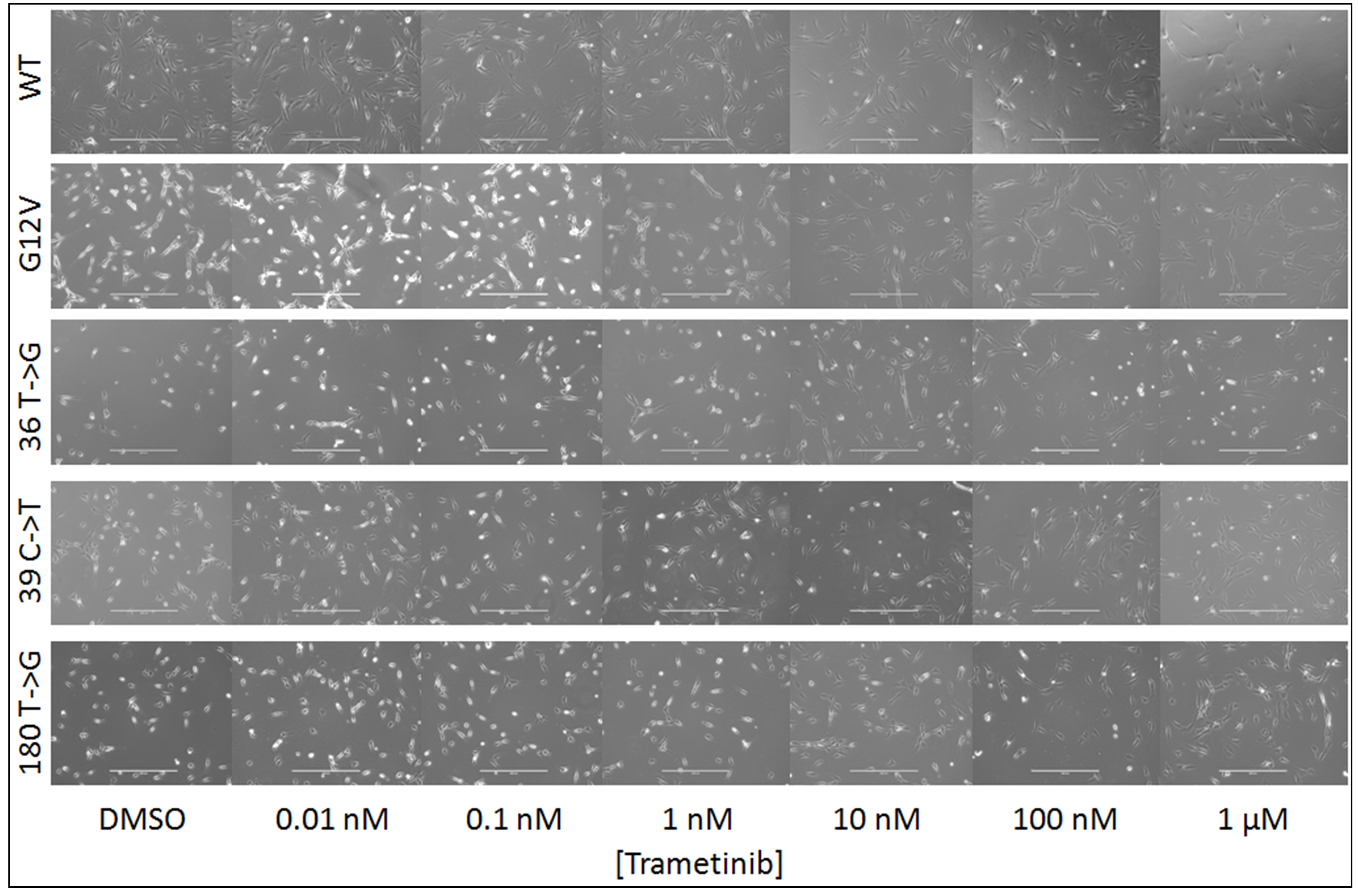

Supplement: S4 Fig — Synonymous mutant cell lines that have lost contact inhibition have a rounded, refractile morphology. With increasing concentrations of trametinib, the cells revert toward a flattened, spread out morphology characteristic of the wild-type cell line. (TIF) [file pone.0163272.s004.tif]

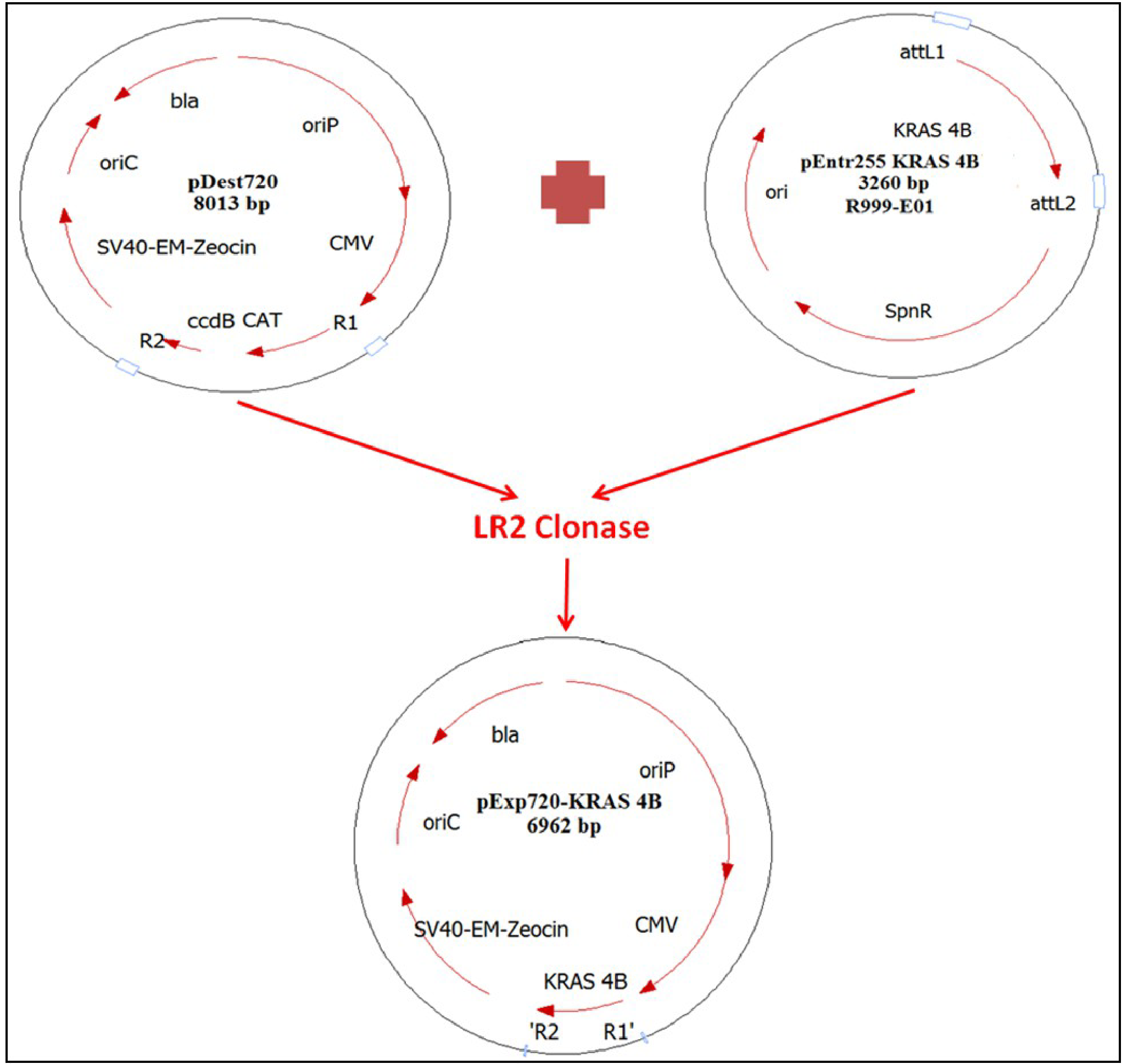

Supplement: S5 Fig — Gateway cloning was employed to replace the ccdB and CAT genes in pDest720 with a KRAS gene. Eleven different KRAS plasmids (WT, G12V, 9 silent mutants) were generated, each containing a unique KRAS gene and a zeocin resistance gene. (TIFF) [file pone.0163272.s005.tiff]

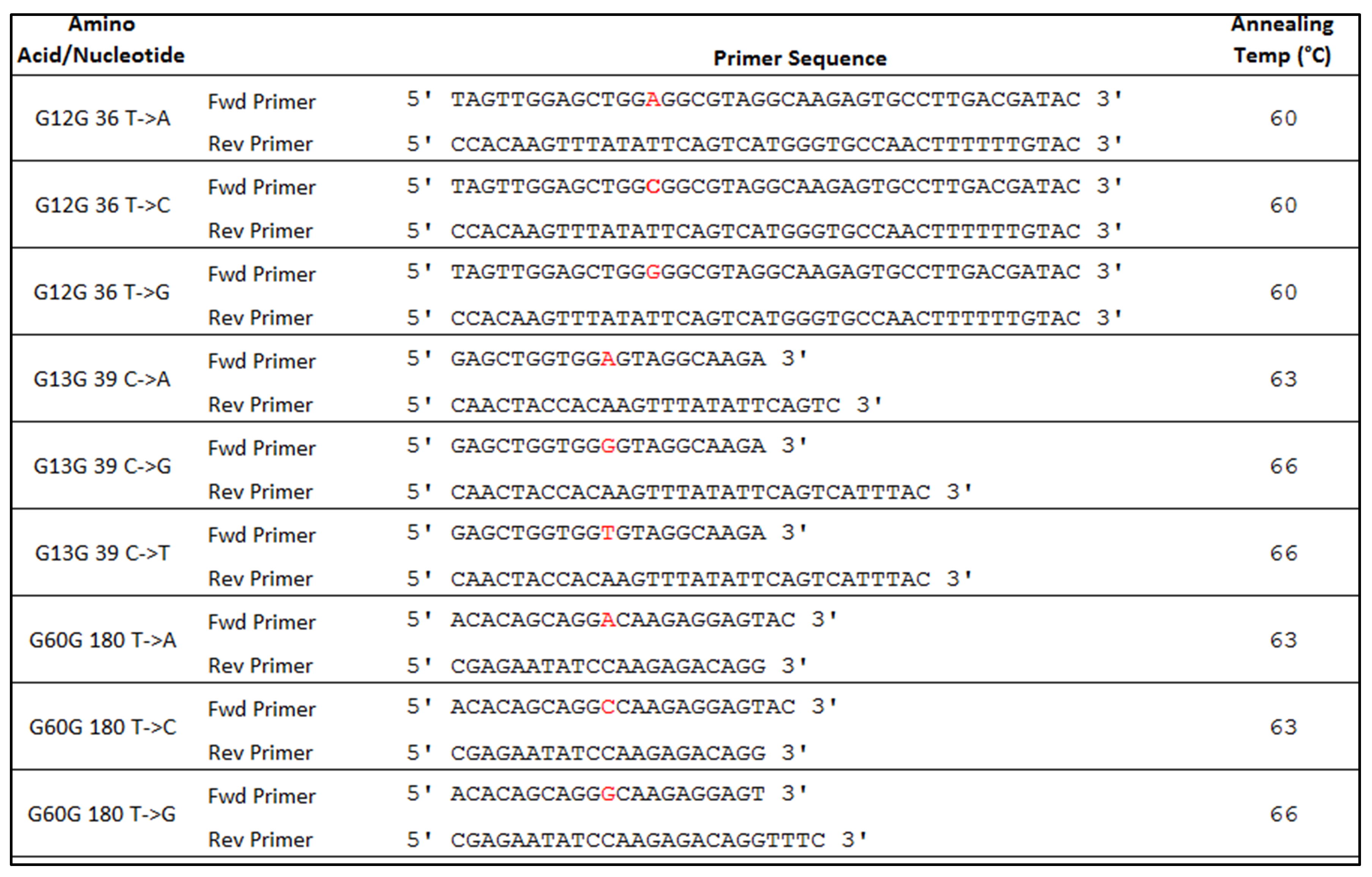

Supplement: S1 Table — (TIF) [file pone.0163272.s008.tif]

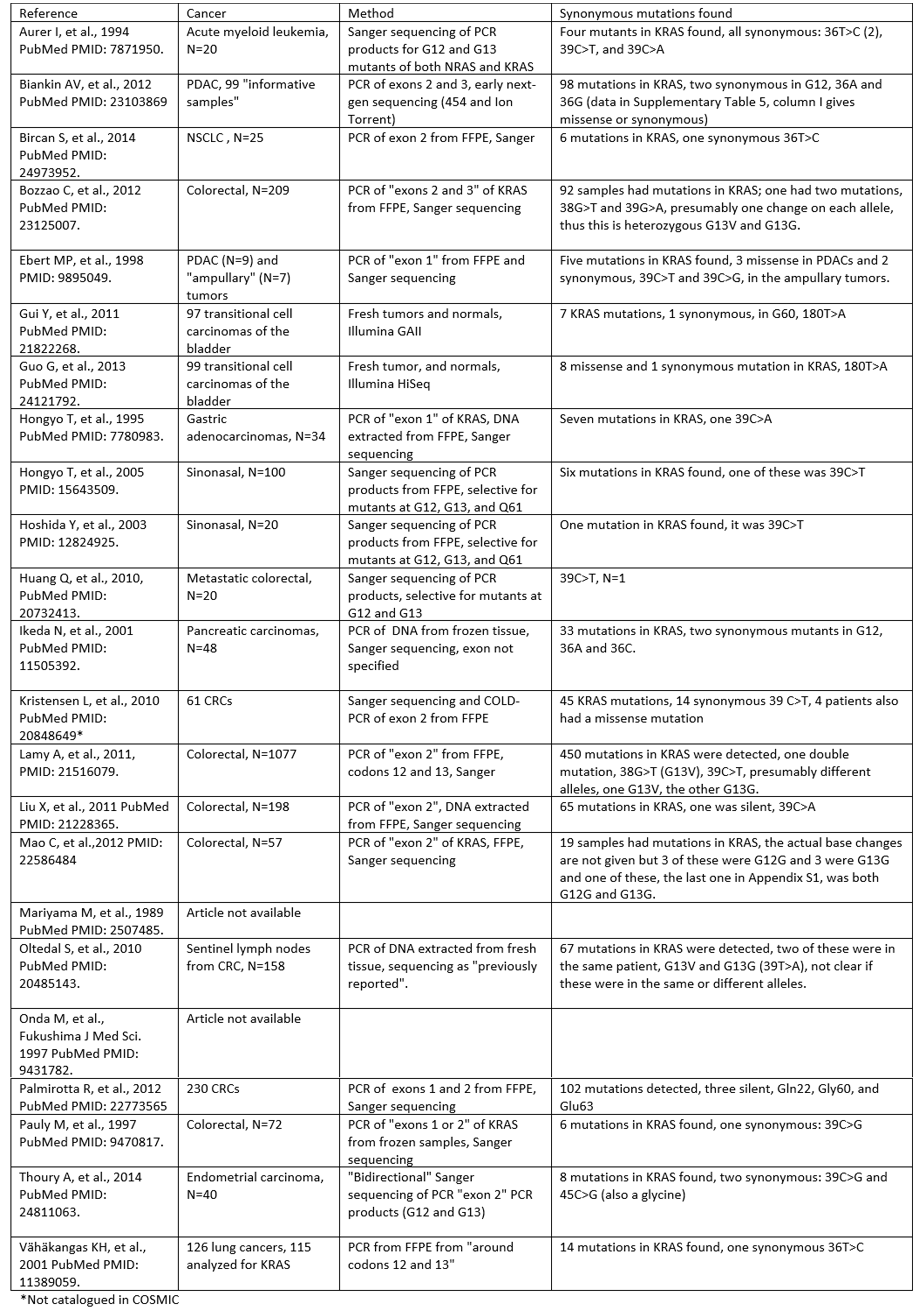

Supplement: S2 Table — (TIF) [file pone.0163272.s009.tif]

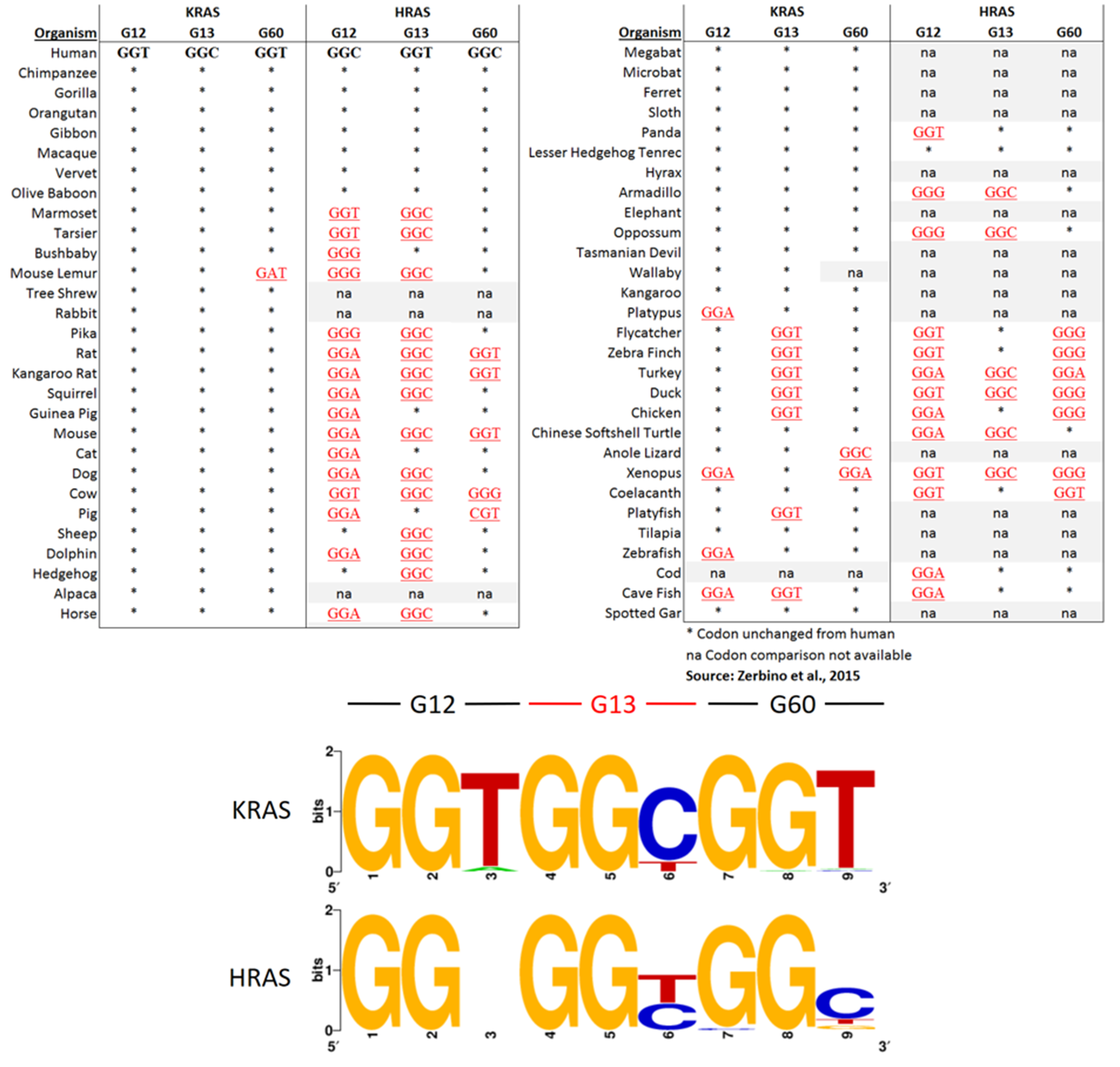

Supplement: S3 Table — DNA sequence logos were constructed according to [45]. (TIFF) [file pone.0163272.s010.tiff]
