# Supplementary material for: Single Synonymous Mutations in KRAS Cause Transformed Phenotypes in NIH3T3 Cells
Source: PLoS One. 2016 Sep 29;11(9):e0163272. doi: 10.1371/journal.pone.0163272 (PMC5042562; doi:10.1371/journal.pone.0163272)
Supplement: S1 File — Methods include mRNA quantification and structure prediction. (DOCX) [file pone.0163272.s006.docx]

**S1 File. mRNA methods.** Methods include mRNA quantification and structure prediction.

**Human KRAS-4B mRNA Quantification**

Total RNA was purified from transiently transfected cells with TriZOL LS (Life Technologies Corp, Carlsbad, CA) and converted to cDNA using the High Capacity cDNA Reverse Transcription Kit (Life Technologies Corp., Carlsbad, CA) according to the manufacturer’s instructions. Droplet-digital PCR was performed on the cDNA samples using the primers listed in ***S1*** ***Table*** using a QX-100 droplet generator and a DG8 cartridge (Bio-Rad, Hercules, CA) according to the manufacturer’s protocol. All values were normalized to GAPDH cDNA levels.

**Prediction Programs**

Secondary structures and free energies of the *KRAS* coding region were predicted by entering the mRNA sequence of each construct, from the start codon to the stop codon, into the MFold web server [31]. To examine the local predicted secondary structure and free energy, the first 61 nucleotides downstream of the start site were used for G12V, G12G and G13G mutations. Nucleotides 150-211 were used to examine the local predicted secondary structure and free energy of the G60G mutations.
